# Identification of biomarkers related to angiogenesis in myocardial ischemia-reperfusion injury and prediction of potential drugs

**DOI:** 10.1371/journal.pone.0300790

**Published:** 2024-06-27

**Authors:** Yaowei Zhao, Hongyu Li, Xiyuan Ma, Xianghong Meng, Qiang Tang

**Affiliations:** 1 Heilongjiang University of Chinese Medicine, Harbin, Heilongjiang, China; 2 Second Affiliated Hospital of Heilongjiang University of Chinese Medicine, Harbin, Heilongjiang, China; National Institutes of Health, UNITED STATES

## Abstract

Myocardial ischemia-reperfusion injury (MIRI) refers to the secondary damage to myocardial tissue that occurs when blood perfusion is rapidly restored following myocardial ischemia. This process often exacerbates the injury to myocardial fiber structure and function. The activation mechanism of angiogenesis is closely related to MIRI and plays a significant role in the occurrence and progression of ischemic injury. In this study, we utilized sequencing data from the GEO database and employed WGCNA, Mfuzz cluster analysis, and protein interaction network to identify Stat3, Rela, and Ubb as hub genes involved in MIRI-angiogenesis. Additionally, the GO and KEGG analysis of differentially expressed genes highlighted their broad participation in inflammatory responses and associated signaling pathways. Moreover, the analysis of sequencing data and hub genes revealed a notable increase in the infiltration ratio of monocytes and activated mast cells. By establishing key cell ROC curves, using independent datasets, and validating the expression of hub genes, we demonstrated their high diagnostic value. Moreover, by scrutinizing single-cell sequencing data alongside trajectory analysis, it has come to light that Stat3 and Rela exhibit predominant expression within Dendritic cells. In contrast, Ubb demonstrates expression across multiple cell types, with all three genes being expressed at distinct stages of cellular development. Lastly, leveraging the CMap database, we predicted potential small molecule compounds for the identified hub genes and validated their binding activity through molecular docking. Ultimately, our research provides valuable evidence and references for the early diagnosis and treatment of MIRI from the perspective of angiogenesis.

## 1. Introduction

Myocardial ischemia-reperfusion injury (MIRI) is characterized by secondary damage to myocardial tissue during the reestablishment of blood flow. Ischemic heart disease has emerged as one of the leading causes of global mortality in recent years, with over 40% of cardiovascular-related deaths in China attributed to it in 2019 [1]. Moreover, in conditions like coronary heart disease and stroke, although the transient ischemia or occlusion of coronary arteries can be resolved through reperfusion, the resulting damage during this process significantly affects the prognosis of patients with myocardial ischemia [2]. The mechanisms underlying MIRI involve the release of oxygen free radicals, intracellular calcium overload, and disruptions in cellular metabolic balance subsequent to the restoration of blood flow and oxygen supply. These mechanisms further compromise the structure and function of myocardial cells, ultimately culminating in myocardial infarction or even sudden death [3–5].

Clinical management of MIRI primarily focuses on controlling short-term episodes of ischemia and reperfusion, employing medication therapy, and utilizing various treatment approaches such as antiplatelet aggregation, anti-inflammatory methods, hypothermia, and electrical stimulation therapy [6]. Through ongoing in-depth research on this disease, a newly recognized form of cell death, primarily driven by ferroptosis, has been identified as a significant participant in regulating the pathological damage process of MIRI. Consequently, research focusing on ferroptosis has revealed that compounds such as resveratrol, naringenin, and ferulic acid can enhance the prognosis of MIRI by upregulating the GPX4 pathway, thereby inhibiting cell ferroptosis and ultimately improving the overall prognosis of MIRI [7–9]. It is noteworthy that, in comparison to the aforementioned treatment strategies, investigating early interventions for myocardial ischemia-reperfusion injury may harbor greater potential and value in attenuating the progression of heart tissue damage.

Angiogenesis, the process of new capillary formation from pre-existing blood vessels under conditions of ischemia and hypoxia, is an essential adaptive response regulated by a variety of physiological and pathological factors [10]. Research indicates that promoting angiogenesis following reperfusion after myocardial infarction can offer short-term protection to the ischemic myocardium, restore physiological function of myocardial cells, and enhance both cardiac blood supply and myocardial function. Additionally, angiogenesis can improve cell survival in the peri-infarct zone, leading to improved ventricular remodeling and the prevention of heart failure [11, 12]. Hence, considering the clinical importance of angiogenesis in the management and prevention of ischemic heart disease, the investigation of early biomarkers for angiogenesis during the myocardial ischemia-reperfusion injury process and the development of therapeutic strategies assumes paramount significance.

In this study, we analyzed gene expression data of MIRI mice obtained from the GEO database using bioinformatics methods. Differential gene expression analysis and weighted gene co-expression network analysis identified angiogenesis-related genes associated with MIRI. Hub genes in early angiogenesis were determined through Mfuzz clustering and protein-protein interaction network analysis, followed by immune infiltration analysis. We validated the identified hub genes through ROC analysis, independent dataset verification, and single-cell sequencing. Additionally, we predicted small molecule compounds targeting these hub genes and analyzed their binding sites through molecular docking, aiming to explore potential therapeutic strategies for MIRI through angiogenesis.

## 2. Materials and methods

### 2.1 Sources of genetic data

We obtained gene sequencing data related to MIRI from the GEO database (https://www.ncbi.nlm.nih.gov/gds). Specifically, we downloaded the gene microarray data of MIRI in mice from the GSE193997 dataset, generated on the GPL17021 platform. This dataset comprises a total of 24 tissue samples, including 3 samples of heart tissue from the control group, 3 samples of ischemic tissue, and 18 samples of ischemia-reperfusion injury tissue.

### 2.2 Screening and analysis of differential genes

In the analysis conducted using R Studio, the dataset underwent a systematic processing protocol. Initially, upon acquiring the raw dataset data, the limma package was employed to normalize gene expression through log2 transformation [13]. Subsequently, a meticulous curation process ensued, involving the elimination of empty and duplicated gene probes to derive a set of maximally informative gene expression probes. Ultimately, the limma package was deployed for the discernment of differentially expressed genes (DEGs), characterized by a log-fold change exceeding 0.3 and a p-value below 0.05. Subsequently, a visual heatmap was generated to illustrate the results. To explore the biological processes (BP), cellular components (CC), and molecular functions (MF) associated with the identified DEGs in myocardial ischemia-reperfusion injury, we employed the "cluster Profiler" package in R software for Gene Ontology (GO) analysis [13]. Furthermore, we conducted KEGG pathway enrichment analysis on the potential genes associated with myocardial ischemia-reperfusion injury using the online database KOBAS 3.0 (http://kobas.cbi.pku.edu.cn) to determine their functional relevance.

### 2.3 Weighted co-expression network analysis

Utilized the WGCNA package in R software to analyze gene expression profiles and construct weighted gene co-expression modules [13]. The correlation between these modules and the control group, as well as the myocardial ischemia-reperfusion injury model group, was examined. The analysis involved calculating gene correlation coefficients and constructing a correlation-based matrix, which was then transformed into an adjacency matrix using power-based weighting. Topological overlap analysis was conducted to determine gene associations and convert the adjacency matrix into a topological matrix. Subsequently, clustering based on node dissimilarity was performed to identify distinct gene modules. Similar modules were merged based on their module eigengenes (ME) as characteristic vectors. Finally, dynamic pruning was employed to partition the modules and a gene dendrogram was generated, with branches and different colors representing individual gene modules.

### 2.4 Angiogenesis related genes download

The MSigDB (https://www.gsea-msigdb.org/gsea/msigdb) houses a comprehensive collection of annotated gene sets. By visiting the official website of MSigDB, researchers can search and download relevant genes by entering the keyword "angiogenesis." Furthermore, genes associated with angiogenesis were extracted from the Genecard database(https://www.genecards.org/). This database primarily encompasses protein sequences derived from whole-genome sequencing of diverse species and includes protein and functional information gathered from literature sources. By comparing the gene lists obtained from both databases, a gene set specifically related to angiogenesis was derived.

### 2.5 Co-expressed genes

Used a Venn diagram to illustrate the overlap of genes among the key modules obtained from WGCNA, the DEGs associated with MIRI, and the gene set related to angiogenesis. This enabled us to visualize the common genes present in all three datasets.

### 2.6 Mfuzz cluster analysis

We performed a time trend analysis using Mfuzz (version 2.6.1, http://www.bioconductor.org/packages/release/bioc/html/Mfuzz.html) to explore the co-expression patterns of genes in MIRI. The GSE193997 dataset provided sequencing data captured at multiple time points. By assessing the gene expression changes across these time points, we applied an acore threshold of 0.6 to classify the results into distinct modules based on their temporal expression patterns. The findings were visualized through gene time clustering trend plots.

### 2.7 Protein interaction network and hub genes

The STRING platform (version 11.0, https://string-db.org/) is a comprehensive database that includes known and predicted protein-protein interactions. To visualize the protein-protein interaction (PPI) network derived from the co-expressed genes, we uploaded the data to the STRING database and imported the results into Cytoscape software for visualization. The hub genes associated with angiogenesis were determined by ranking the top three genes using the MCC algorithm.

### 2.8 Immune infiltration analysis

The CIBERSORT algorithm was utilized to calculate the proportions of 22 immune cell types in the samples, considering a significance criterion of P < 0.05 [14]. The results were visualized accordingly. Additionally, an immune infiltration analysis was conducted to examine the immune cell composition between the myocardial ischemia-reperfusion injury samples and the control group. Furthermore, a detailed analysis was performed to investigate the correlation between hub genes and different immune cell types.

### 2.9 Evaluation of ROC diagnostic value

The gene expression profiles of GSE108940 and GSE214122 were downloaded. ROC curves were generated using the "pROC" R package to analyze the specific expression of hub genes in the validation set and evaluate their diagnostic value [15]. The area under the ROC curve (AUC) represents the sensitivity and specificity of the genes, indicating the accuracy and reliability of the diagnostic assessment. A higher AUC value, closer to 1, indicates a greater diagnostic value of the gene.

### 2.10 Hub gene expression verification

To further validate the screening results of hub genes associated with angiogenesis, we obtained the sequencing data of GSE83472 as a validation dataset. The expression of these hub genes was examined to ensure the accuracy of the bioinformatics analysis results.

### 2.11 Single-cell transcriptomics and trajectory analysis

Downloaded the dataset GSE227238 and performed an analysis using the Seurat package [16]. Utilized t-distributed stochastic neighbor embedding (t-SNE) for clustering and dimension reduction analysis, with a specific focus on the expression profiles of prognostic genes within distinct clusters. Employed the SingleR package for annotating diverse cell subpopulations [16]. Additionally, conducted trajectory analysis of cell developmental stages using the Monocle package to elucidate the expression patterns of Hub genes across cells at various developmental stages [16].

### 2.12 Prediction of hub gene drugs

Hub genes were inputted into both the Connectivity Map (CMap) database (https://portals.broadinstitute.org/cmap) and the GeneCard database to establish associations between drugs, genes, and diseases. Subsequently, a screening process was performed to identify small-molecule compounds that are linked to the hub genes associated with angiogenesis.

### 2.13 Molecular docking of hub genes and small molecule compounds

The 3D structures of the hub genes were retrieved from the PDB database (https://www.rcsb.org/), while the structures of small-molecule compounds were obtained from Drugbank(https://www.drugbank.com/). The target proteins underwent several operations, including desolvation and hydrogenation, using PyMOL software. Docking between the hub genes and small-molecule compounds was conducted using the CB-Dock2 database.

## 3. Results

### 3.1 Differentially expressed genes in myocardial ischemia-reperfusion injury

**Fig 1:**
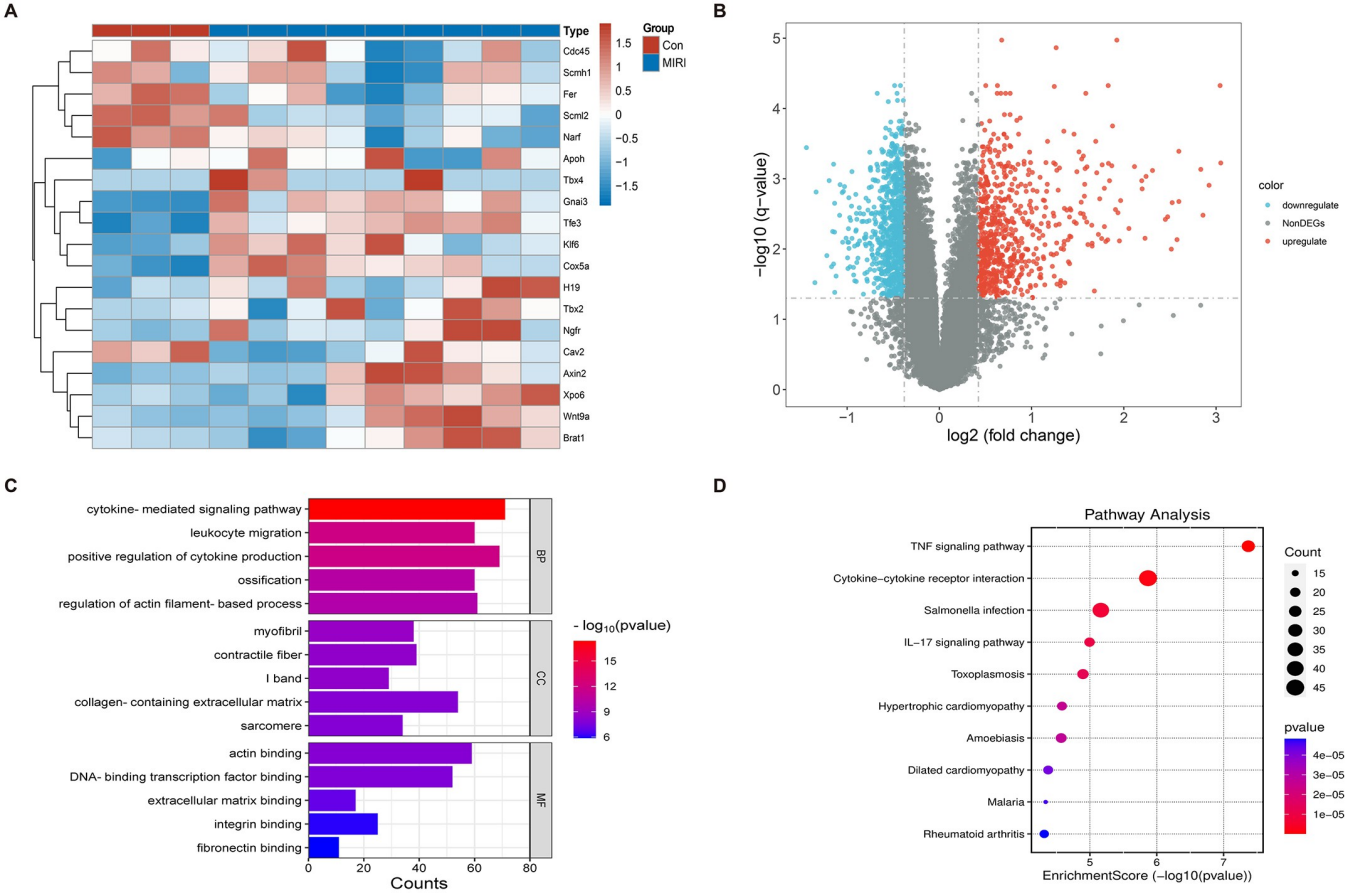
Analysis of DEGs in myocardial ischemia-reperfusion injury. (A) Heatmap of gene expression in the dataset. (B) Volcano plot of differentially expressed genes, with red indicating upregulated genes and blue indicating downregulated genes. (C) GO enrichment analysis of differentially expressed genes. (D) KEGG enrichment analysis of differentially expressed genes.

Analysis of the GSE193997 dataset identified a total of 1528 DEGs by applying the specified filtering criteria. Among these DEGs, 708 genes were upregulated while 820 genes were downregulated (Fig 1A and 1B). GO functional enrichment analysis indicated that the DEGs were primarily localized in myofibrils, contractile fibers, I bands, collagen-containing extracellular matrix, and sarcomeres. These DEGs were found to be involved in various biological processes, including leukocyte migration, positive regulation of cytokine production, ossification, and regulation of actin filament-based processes. Moreover, they were implicated in key signaling pathways, such as the TNF signaling pathway, Cytokine-cytokine receptor interaction, and IL-17 signaling pathway (Fig 1C). KEGG pathway analysis further highlighted the enrichment of DEGs in important signaling pathways, including the TNF signaling pathway, Cytokine-cytokine receptor interaction, and IL-17 signaling pathway (Fig 1D). Consult Supplementary Material 1 in S1 File for a detailed presentation of the analysis results for both GO and KEGG.

### 3.2 Screening of modules related to myocardial ischemia-reperfusion injury

**Fig 2:**
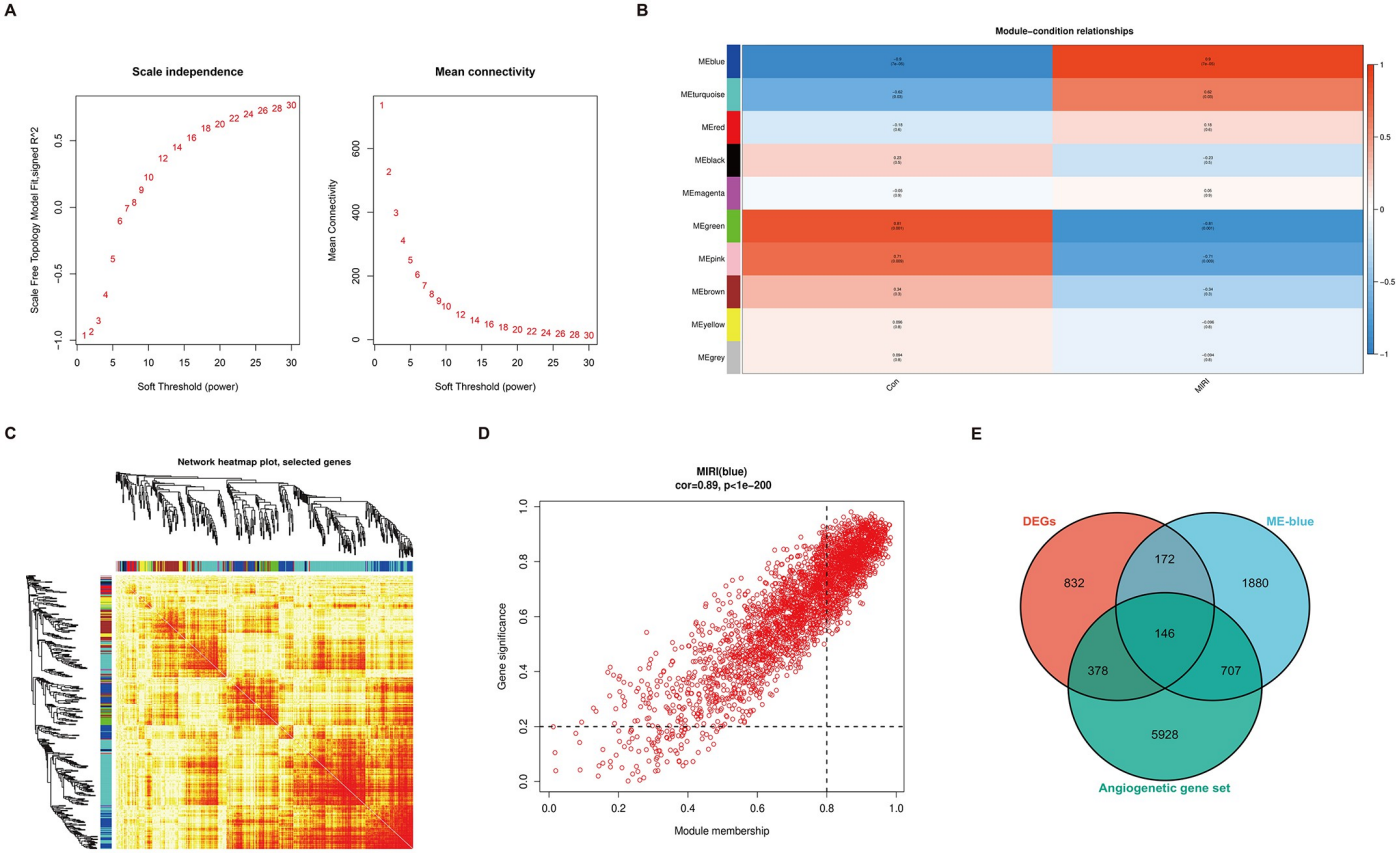
MIRI-WGCNA related modules and gene screening. (A) Determining the soft threshold. (B) Heatmap depicting the correlations among MIRI-related genes. (C) Gene co-expression network heat map, the left and upper sides of the figure are the results of symmetric phylogenetic clustering trees and gene modules; the lower right heat map area represents the dissimilarity between genes, the smaller the value, the darker the color. (D) Scatterplot of module assignment and gene significance. (E) Intersection results of MEblue, DEGs and angiogenesis genes.

The results of the WGCNA indicated that by increasing the soft-thresholding power to calculate the scale-free topology fit index and average connectivity, a more favorable connectivity relationship was achieved with a soft-thresholding power (β) set to 22 (Fig 2A). Subsequently, the correlation between modules and groups was computed, and a correlation heatmap was generated. The blue module showed a high correlation value of 0.9 with the MIRI group, suggesting a strong positive correlation between the genes within this module and MIRI (Fig 2B). Additionally, the connectivity of genes between different modules was assessed, and the results demonstrated a well-defined module division effect for WGCNA (Fig 2C). Following this, an investigation into the association between genes within the blue module and MIRI was carried out by calculating the correlation coefficients. The findings revealed a significant positive correlation (cor = 0.89, P < 0.05) between the MEblue and MIRI (Fig 2D). Subsequently, the genes within the MEblue were overlapped with DEGs and the gene set related to angiogenesis, leading to the identification of a final set of 146 co- expression genes (Fig 2E).

### 3.3 Mfuzz cluster analysis of co-expressed genes

**Fig 3:**
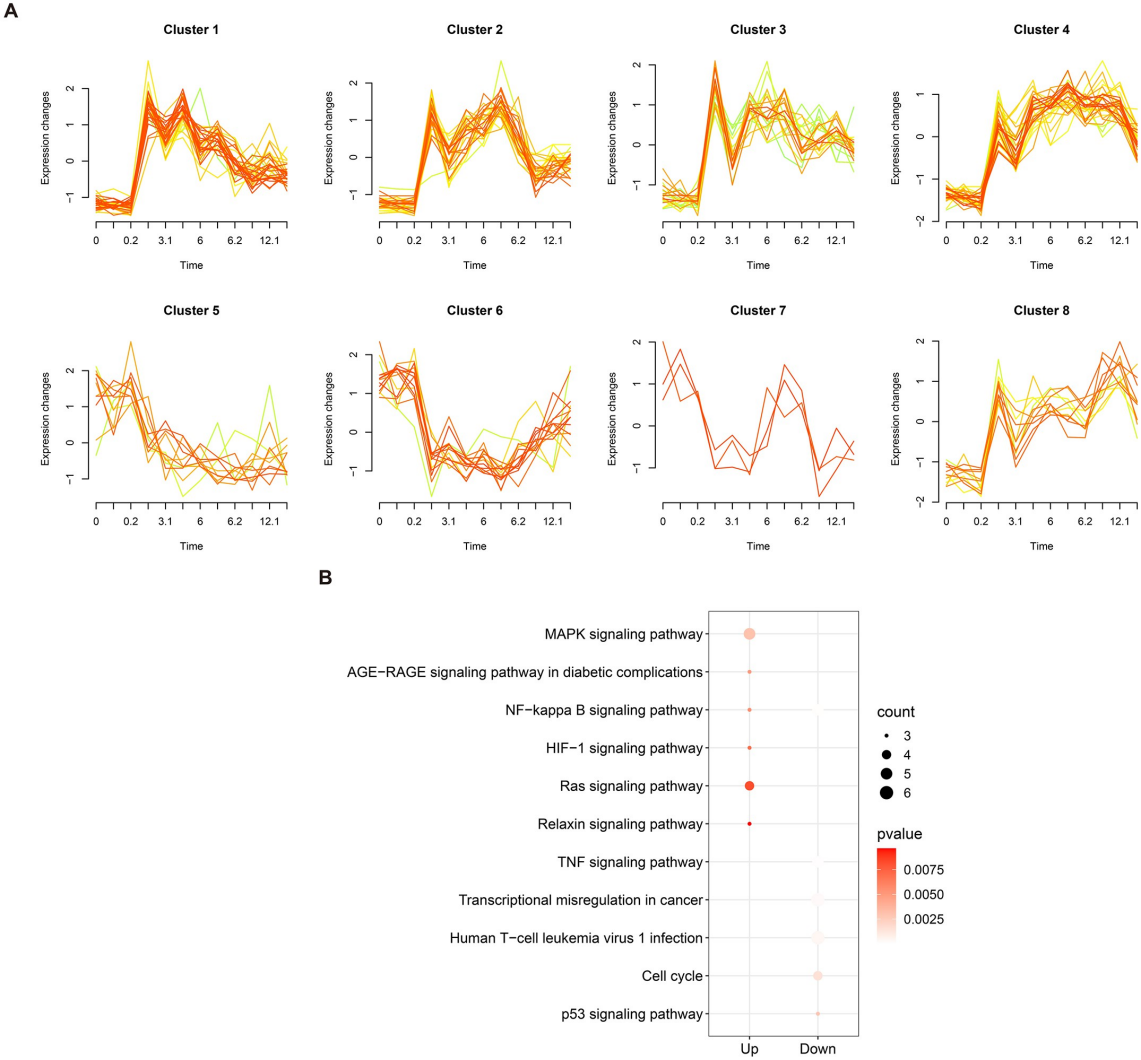
Mfuzz cluster analysis. (A) Cluster analysis of the trend of co-expressed genes in 8 clusters: the X-axis represents time, while the Y-axis represents gene expression. (B) KEGG enrichment analysis of up-regulated and down-regulated cluster genes.

The co-expressed genes was analyzed using the Mfuzz software for time series analysis, resulting in the formation of 8 gene clusters. Over time, these gene clusters exhibited distinct patterns of change. Notably, Cluster 1, Cluster 2, Cluster 3, and Cluster 7 displayed significant fluctuations in gene expression, while Cluster 5 and Cluster 6 showed a decreasing trend. Conversely, Cluster 4 and Cluster 8 exhibited an increasing trend (Fig 3A). Further pathway analysis revealed that the upregulated genes were primarily enriched in signaling pathways such as MAPK signaling pathway, AGE-RAGE signaling pathway, NF-kappa B signaling pathway, HIF-1 signaling pathway, and Ras signaling pathway. On the other hand, the downregulated genes were predominantly associated with pathways such as TNF signaling pathway and p53 signaling pathway (Fig 3B).

### 3.4 Protein- protein interaction

**Fig 4:**
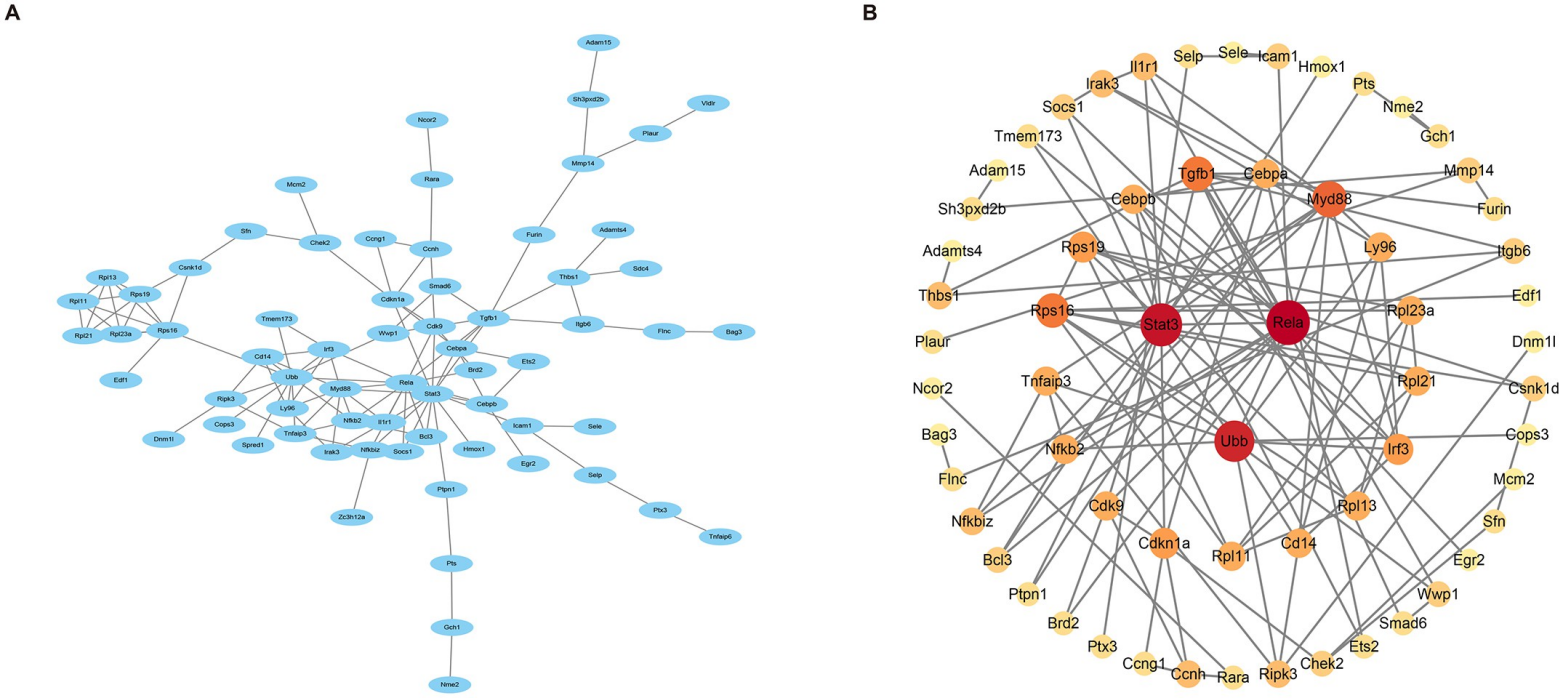
Analysis of co-expressed gene-protein interaction and identification of hub genes. (A) Protein interaction network. (B) Visualization of hub genes.

Having excluded two unidentified proteins, the network graph, comprising 144 nodes and 368 interactions, was derived from the analysis conducted in the STRING database. Subsequently, the network graph was imported into the Cytoscape software for in-depth examination (Fig 4A). To assess the strength of interactions within the protein-protein interaction (PPI) network, the MCODE plugin in Cytoscape was employed. Notably, stronger interactions between nodes were visually represented by darker colors and larger node sizes. Employing the MCC algorithm, the top three hub genes associated with angiogenesis were identified as Stat3, Rela, and Ubb (Fig 4B).

### 3.5 Immune infiltration analysis

**Fig 5:**
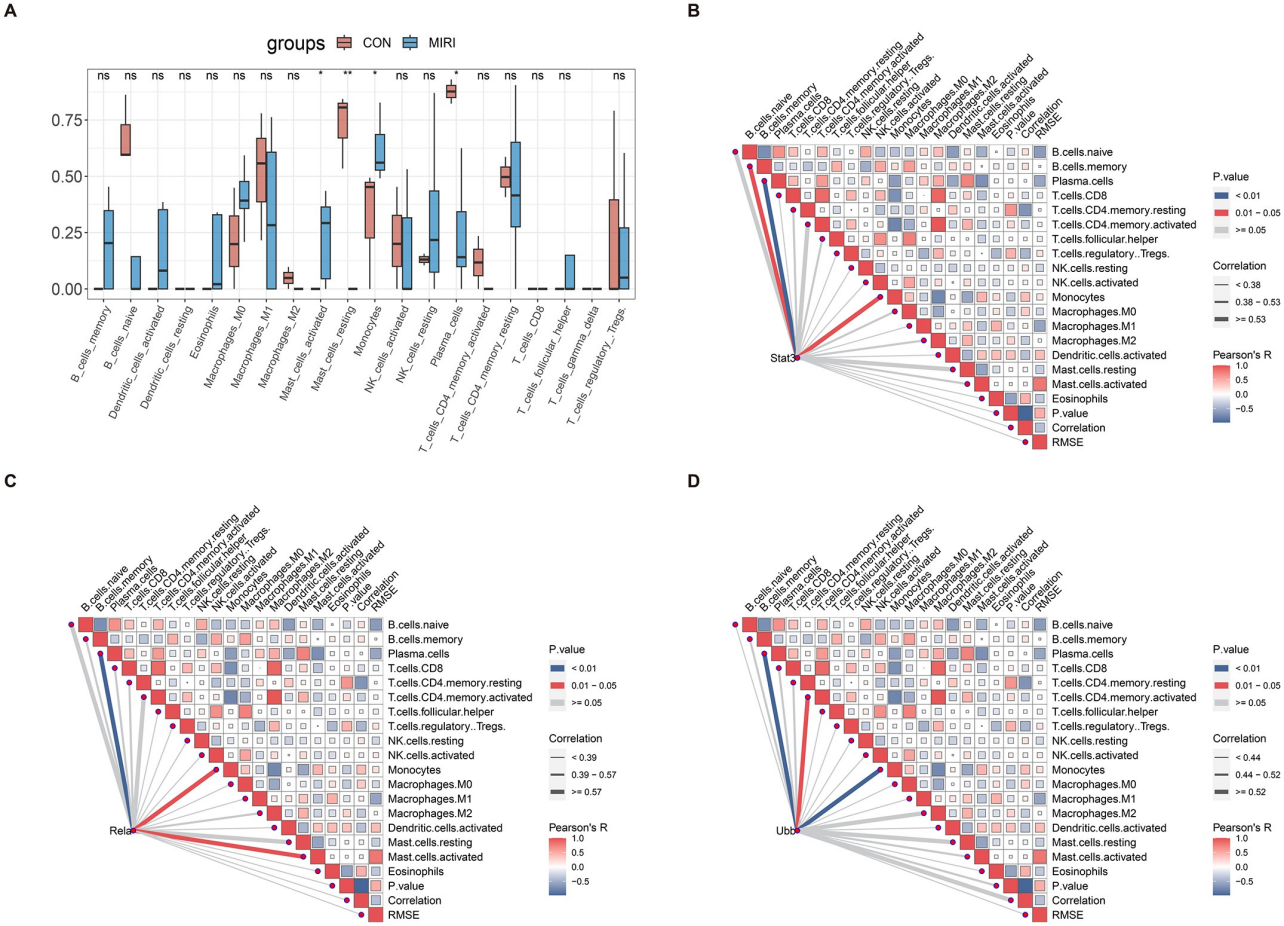
Correlation analysis of immune infiltration and Hub gene immune infiltration. (A) The infiltration of various immune cell types in the GSE193997 dataset, red and blue represent the control group and myocardial ischemia-reperfusion injury group, respectively. (B) Stat3 (C) Rela (D) Ubb.

The results of DEGs enrichment analysis (GO and KEGG) and co-expression gene clustering analysis (Mfuzz) indicate a significant correlation between MIRI, angiogenesis, and immune response. To further investigate this relationship, CIBERSORT was employed to analyze the infiltration of immune cells in the expression profiles of MIRI and control groups. The analysis revealed noteworthy disparities in the infiltration patterns of 22 immune cell types between MIRI and normal myocardial tissues. Specifically, monocytes and activated mast cells exhibited significantly higher infiltration proportions in the MIRI group compared to the normal group, while resting mast cells and plasma cells displayed the opposite trend (Fig 5A). In terms of specific gene associations, Stat3 showed the strongest correlation with plasma cells, followed by memory B cells and monocytes. Rela demonstrated the highest association with plasma cells, followed by activated mast cells. Ubb exhibited the strongest association with plasma cells and monocytes, followed by activated memory CD4+ T cells (Fig 5B–5D). For the comprehensive expression profile of CIBERSORT, kindly consult Supplementary Material 2 in S1 File.

### 3.6 ROC diagnostic value of hub genes

**Fig 6:**
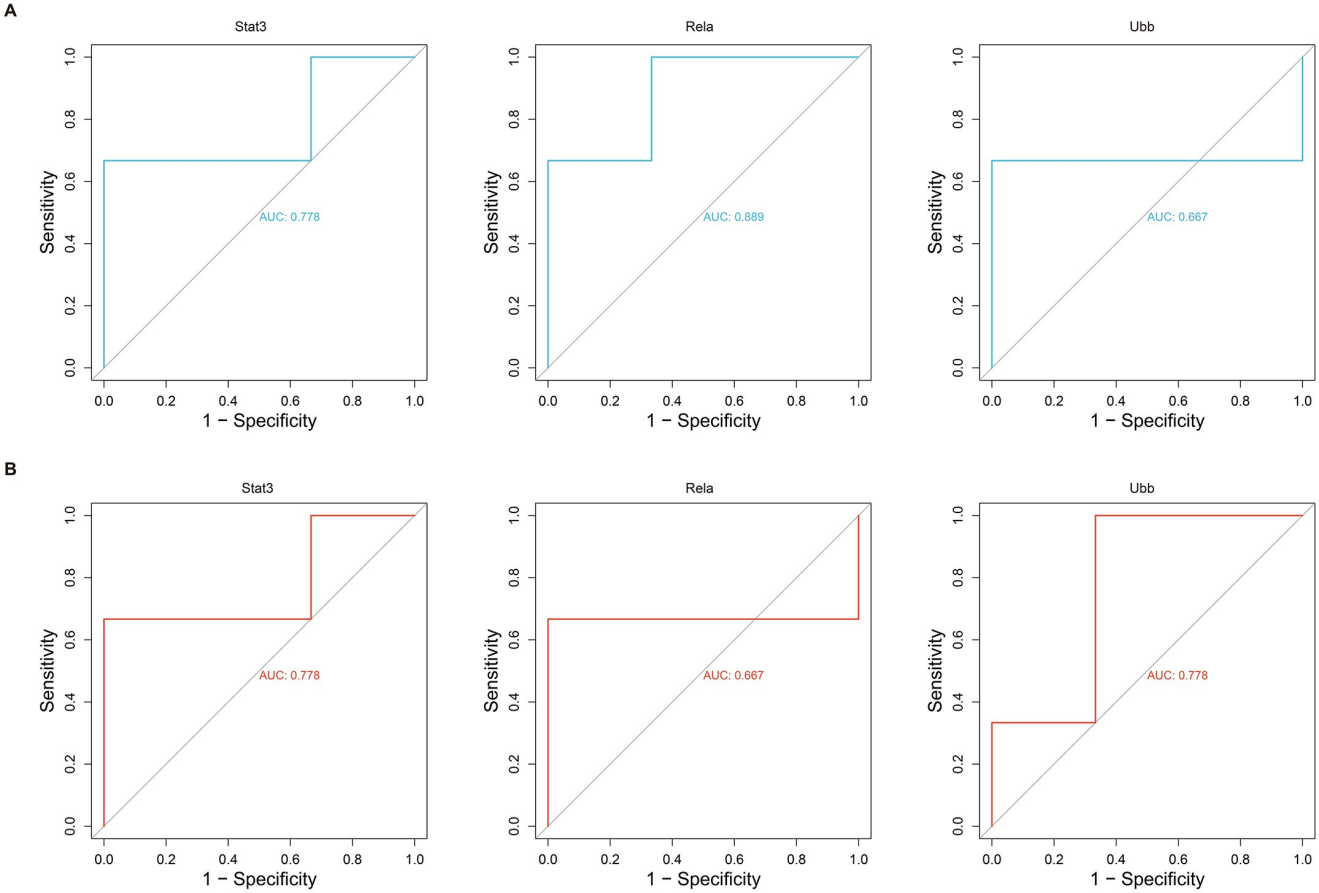
Hub gene ROC curves. (A) ROC curves of hub genes in the GSE214122 dataset. (B) ROC curves of hub genes in the GSE108940 dataset.

The expression profiles from the GEO databases GSE214122 and GSE108940 were downloaded to perform ROC curve analysis and evaluate the diagnostic value of the hub genes (Fig 6). The results showed that Stat3 had a moderate level of diagnostic accuracy in both expression profiles (AUC = 0.778, AUC = 0.778). Rela demonstrated reasonable diagnostic accuracy in the GSE214122 expression profile (AUC = 0.889), but its diagnostic accuracy was lower in the GSE108940 dataset (AUC = 0.667). Ubb exhibited lower diagnostic accuracy in the GSE214122 dataset (AUC = 0.667), whereas it showed a certain level of accuracy in the GSE108940 expression profile (AUC = 0.778). These findings suggest that three pivotal genes associated with angiogenesis exhibit significant diagnostic efficacy and reliability.

### 3.7 Hub gene expression verification

**Fig 7:**
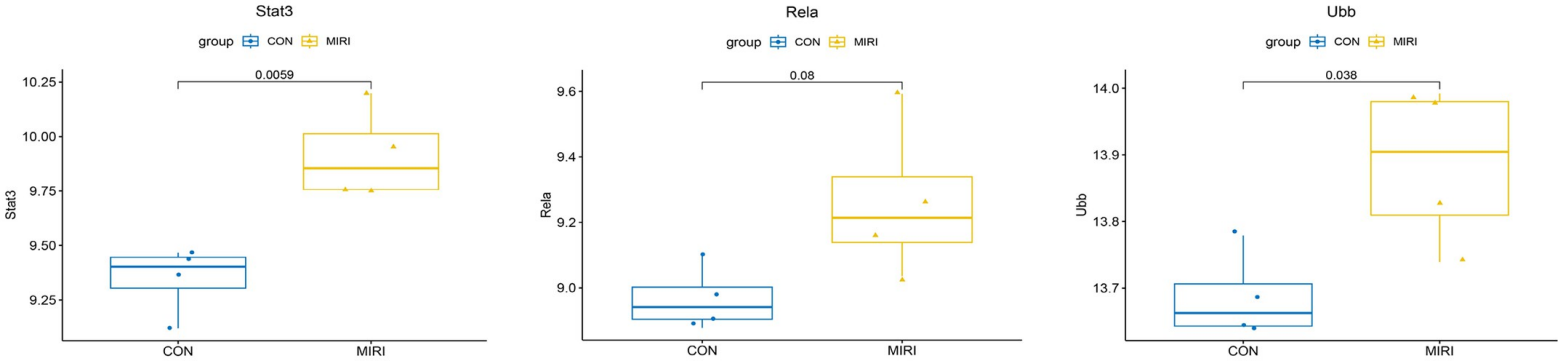
Hub gene independent data set expression validation.

The expression profiles from the GEO database were downloaded, and GSE83472 was utilized as an independent dataset to validate the expression of Stat3, Rela, and Ubb as hub genes. The results revealed that, in comparison to the control group of normal myocardial tissue, the expression of Stat3 was significantly upregulated in MIRI myocardial tissue (*P*<0.01), and the expression level of Ubb was significantly elevated (*P*<0.05). However, despite an increase in expression, Rela did not show statistical significance (Fig 7). Considering the biological variability, sample heterogeneity, and diverse experimental designs between the original set and the validation set, these factors could potentially have influenced the results. This influence might account for the observed elevation in Rela expression during validation, despite the absence of statistical significance.

### 3.8 Single-cell transcriptome and trajectory analysis

**Fig 8:**
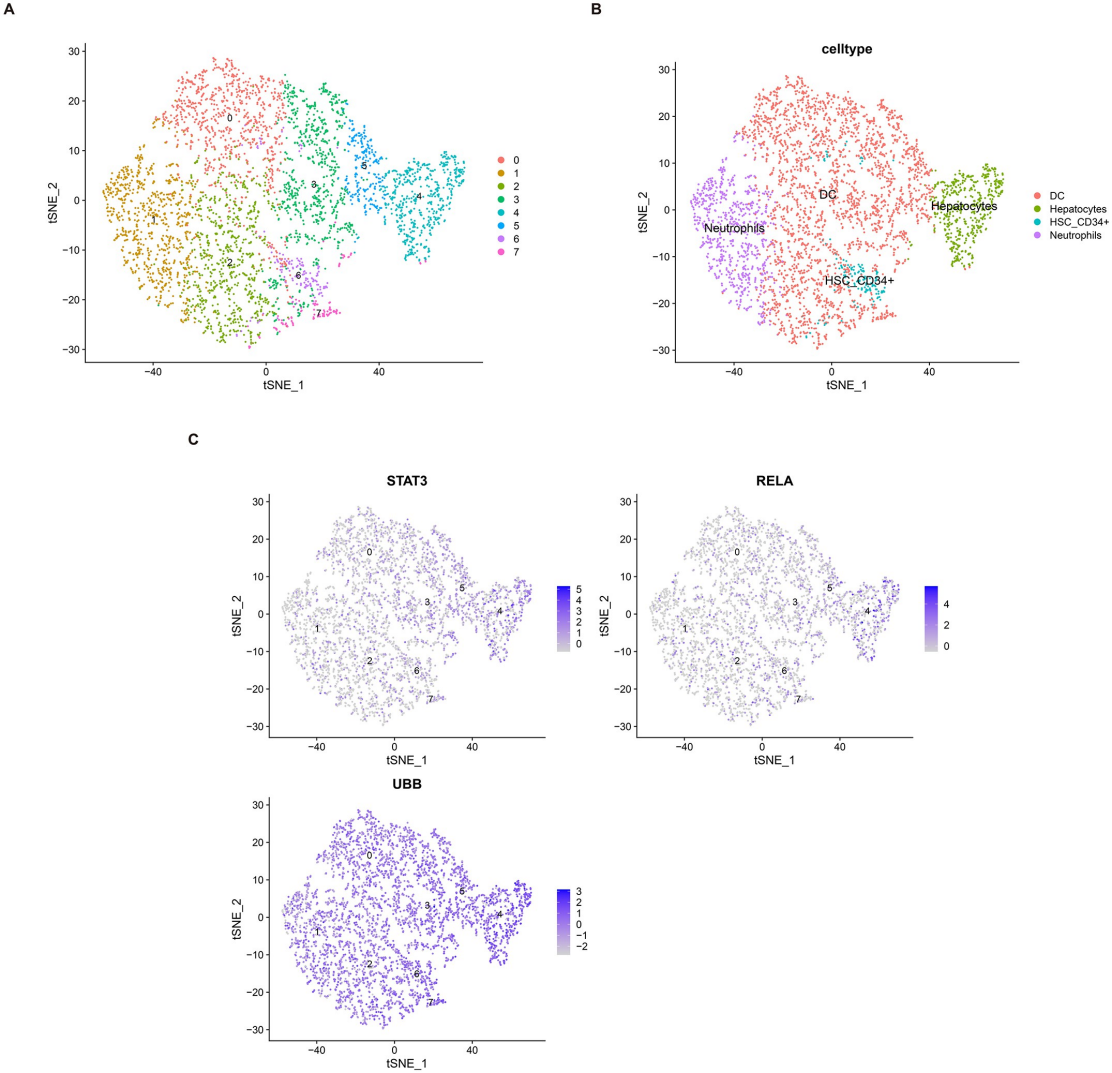
Single-cell RNA sequencing transcriptome Atlas. (A) Visualization of major cell lineages using t-SNE. (B) Identification and annotation of distinct cell lineage types. (C) Analysis of the distribution of hub genes within the single-cell dataset.

Utilizing the Seurat package and performing t-SNE dimension reduction and clustering, we annotated cell subpopulations using the SingleR package, resulting in the identification of four cell subgroups: Dendritic cells, Hepatocytes, HSC-CD34+, and Neutrophils (Fig 8A). Within these cell subgroups, Stat3 and Rela were predominantly expressed in Dendritic cells and Hepatocytes, while Ubb exhibited expression across multiple cell types (Fig 8B).

**Fig 9:**
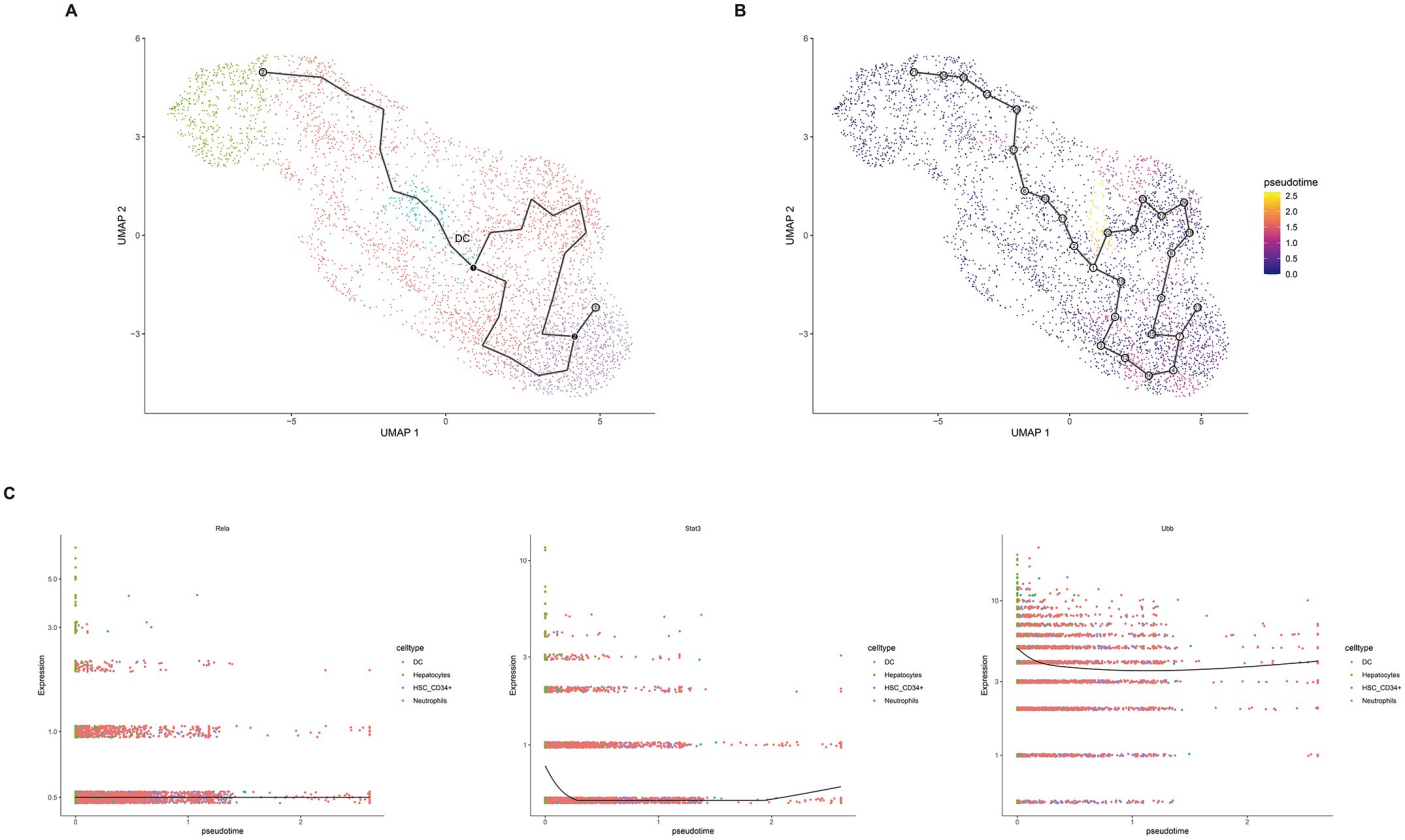
Pseudotime trajectory analysis of scRNA-seq data. (A) Visualization of the single-cell trajectory using UMAP. (B) Calculation of pseudotime-based cell ordering with Monocle3, featuring a color gradient transitioning from purple to yellow. (C) Examination of the expression patterns of hub genes along the pseudotime trajectory.

To further explore potential connections between different stages of cell development along the trajectory and the expression changes of Hub genes, we conducted developmental trajectory and pseudo-temporal analysis using Monocle3. Initially, we performed dimension reduction and visualization analysis using UMAP and subsequently constructed cell trajectories based on the results of pseudo-temporal analysis. Our analysis revealed the existence of two distinct cell trajectories within Dendritic cells (Fig 9A and 9B). The expression patterns of Hub genes in the results of the pseudo-temporal analysis indicated stable expression of Rela, along with consistent expression trends for Stat3 and Ubb (Fig 9).

### 3.9 Molecular docking

**Fig 10:**
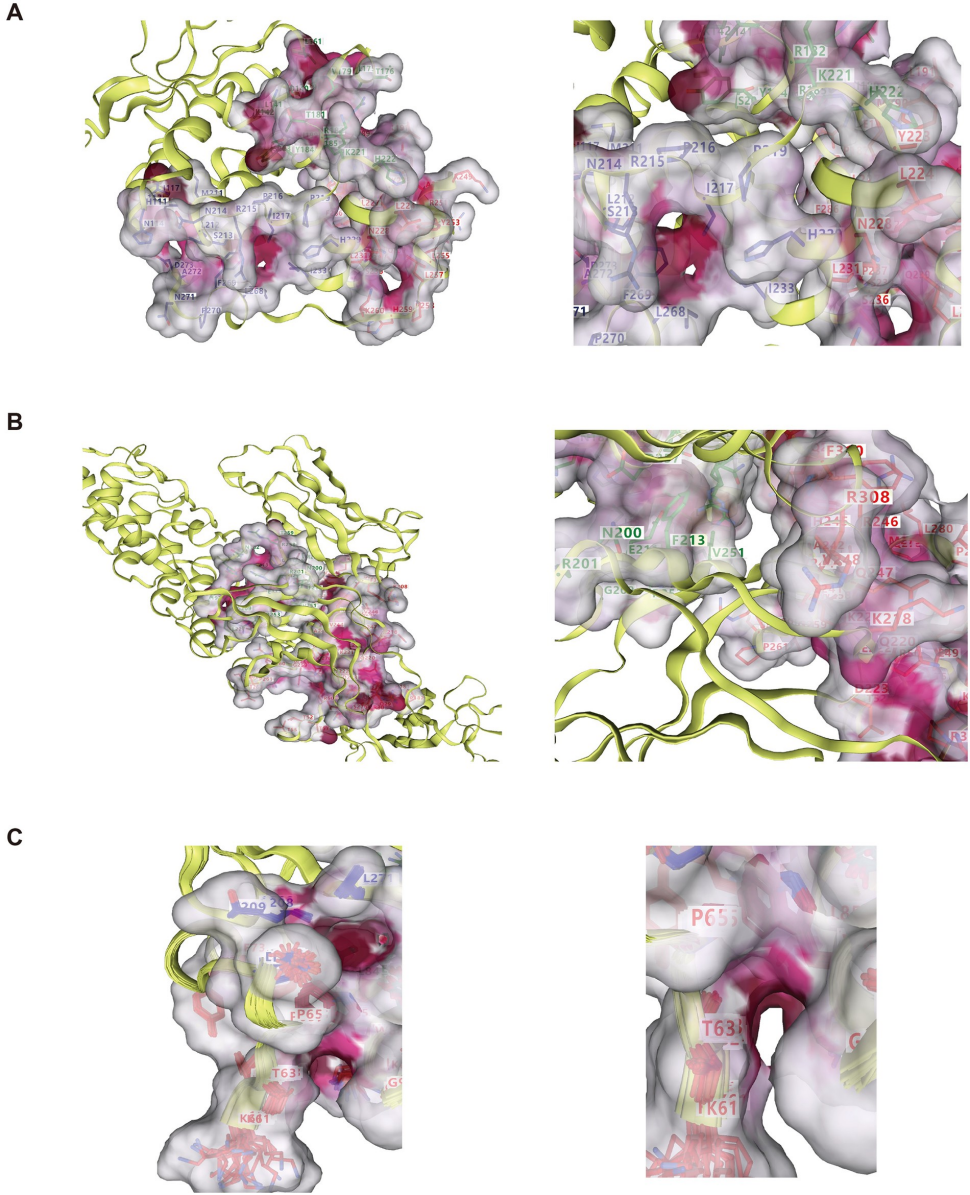
Hub gene-compound molecular docking results. (A) Stat3 (B) Rela (C) Ubb.

The CMap analysis revealed itraconazole and bortezomib as potential compounds for Stat3 and Rela, respectively. We employed GeneCard to identify the potential compound for Ubb, which turned out to be (4s)-5-Fluoro-L-Leucine. To evaluate the affinity between candidate small molecule compounds and their respective targets, we conducted molecular docking analysis (Fig 10). The results of this analysis indicate that the binding energies of Stat3, Rela, and their corresponding potential compounds, itraconazole and bortezomib, are all below -7 kcal·mol-1. These findings suggest that the candidate small molecule compounds exhibit binding activity with their protein targets, and the binding interactions appear to be relatively stable. However, it is unfortunate that Ubb shows a lower binding energy of -2.948 kcal·mol-1 with its potential compound (4s)-5-Fluoro-L-Leucine.

## 4. Discussion

The pathophysiological process of MIRI is notably intricate. It involves the interplay of multiple pathological factors, such as oxidative stress, immune response, energy metabolism imbalance, and intracellular electrolyte disturbance, which collectively contribute to the mediation of MIRI. Consequently, these pathological factors synergistically lead to irreversible damage to myocardial cells, resulting in a sharp deterioration of cardiac function and even precipitating heart failure [17]. In recent years, bioinformatics has emerged as a crucial tool for identifying potential biomarkers and their corresponding drug targets. Among these methods, WGCNA stands out as an effective systems biology approach for investigating the intricate relationships between genes and phenotypes [18]. Primarily designed for the analysis of large-scale gene expression data, WGCNA facilitates the identification of biomarkers, which, in turn, aids in disease diagnosis, drug development, and precision treatment.

In this study, we initially conducted differential gene expression analysis on the gene expression profiles of mice subjected to MIRI. Subsequently, we employed WGCNA to identify the most relevant module associated with MIRI within the expression profiles. We then performed an intersection analysis involving the DEGs, MIRI-related modules, and a set of genes related to angiogenesis to identify co-expressed genes. Utilizing Mfuzz clustering analysis and protein-protein interaction networks, we identified Stat3, Rela, and Ubb as hub genes involved in angiogenesis and conducted an immune infiltration analysis. Moreover, we generated ROC curves and validated them using independent datasets. Additionally, we analyzed the datasets using single-cell sequencing platforms to predict potential small molecule compounds that target the hub genes Stat3, Rela, and Ubb. To further investigate their potential interactions, molecular docking was employed to uncover their binding sites.

DEGs analysis of the gene expression profiles in MIRI mice identified a total of 1528 DEGs. Functional enrichment analysis revealed their predominant involvement in fibrous tissue, leukocyte migration, and cytokine regulation. These DEGs were found to be enriched in inflammatory signaling pathways, including the TNF signaling pathway and IL-17 signaling pathway. KEGG analysis further demonstrated their association with pathological processes related to myocardial injury and inflammation. Despite the complex pathogenesis of MIRI, inflammatory factors play a pivotal role in its development. Ischemia-reperfusion of myocardial tissue triggers the release of pro-inflammatory factors, such as TNF-α and IL-6, by neutrophils, macrophages, and other cells, leading to vascular occlusion. Moreover, these inflammatory factors are involved in the regulation of TNF-α signaling pathways, IL-17 signaling pathways, and the promotion of peroxidase synthesis. These processes contribute to an accelerated inflammatory response and exacerbation of myocardial injury [19, 20]. In the analysis of the most relevant module in WGCNA, a total of 2905 genes were identified. By overlapping the DEGs with the genes within the relevant module and a set of angiogenesis-related genes, we obtained 146 co-expressed genes. These co-expressed genes were further subjected to Mfuzz trend clustering analysis to determine their temporal expression patterns. Pathway analysis revealed that the genes in the upregulated and downregulated gene clusters were primarily enriched in signaling pathways associated with inflammatory response. Previous studies have demonstrated that these overlapping genes, enriched in inflammatory signaling pathways, play a significant role in the pathophysiological processes of MIRI [21–23]. Finally, a protein-protein interaction network was constructed to validate the involvement of Stat3, Rela, and Ubb as hub genes in angiogenesis during the progression of myocardial ischemia-reperfusion injury. These genes will be further investigated in the subsequent phase of this research.

Angiogenesis is a complex process involving the activation, proliferation, and migration of vascular endothelial cells to form new blood vessel networks, which is regulated by a balance of pro-angiogenic and inhibitory factors. The findings of DEG analysis and Mfuzz trend clustering analysis in this study align with the association between angiogenesis and inflammatory signaling pathways. During transient ischemia and hypoxia in myocardial tissue, the injured tissue exhibits a substantial upregulation of inflammatory cytokines (such as TNF-α, IL-17) and hypoxia-inducible factor (HIF)-1α, leading to the activation of the nuclear transcription factor nuclear factor kappa B (NF-κB) inflammatory pathway and the subsequent induction of vascular endothelial growth factor (VEGF) production and secretion [24]. VEGF, a highly specific pro-angiogenic factor for endothelial cell growth, not only binds to receptors on endothelial cell membranes under hypoxic conditions to stimulate endothelial cell proliferation, but also regulates the chemokine ligand chemokine ligand, CXCL 12, and the chemokine receptor chemokine receptor, CXCR 4, axis, which plays a role in vascular network formation [25, 26]. Therefore, the exploration of potential targets involved in angiogenesis in the context of myocardial ischemia-reperfusion injury holds significant value.

Signal transducer and activator of transcription (STAT) proteins belong to a class of DNA-binding proteins that function as both signal transducers and transcription factors. Among them, STAT3, as a member of the STAT family, plays a crucial role in information transmission by integrating and transducing signals from more than 40 different cytokines and growth factors [27]. The activation and inhibition of STAT3 directly regulate the expression of VEGF, thereby actively participating in angiogenesis. For instance, elevated STAT3 expression can upregulate VEGF expression, promoting angiogenesis, while suppressing STAT3 leads to a decrease in VEGF expression, ultimately inhibiting angiogenesis [28]. Importantly, STAT3 can also stimulate the regeneration of small blood vessels in ischemic myocardial tissue through various signaling pathways. In the case of reduced coronary blood flow, STAT3 can increase the expression levels of HIF-1α via the STAT3/HIF-1α signaling pathway, inducing angiogenesis in the ischemic myocardial tissue. Conversely, patients with Job’s syndrome experience reduced coronary artery perfusion due to defects in the STAT3/HIF-1α signaling pathway [27]. Furthermore, the IL-6/STAT3 signaling pathway is implicated in the prognosis of MIRI. During MIRI, there is a significant increase in the expression of IL-6 and its receptor, which promote angiogenesis through the STAT3 signaling pathway, thereby protecting the injured myocardial tissue [29, 30]. STAT3 is extensively involved in cell proliferation, differentiation, and inflammatory responses. In cancer research, activated p-STAT3 has been observed to modulate B lymphocytes and facilitate tumor progression. In a study on rats with heart failure, Stat3 was found to regulate monocyte chemotaxis, thereby contributing to the progression of heart failure. Reducing the level of STAT3 phosphorylation can inhibit monocyte migration, thereby exerting anti-atherosclerotic effects [31–34]. Immuno-infiltration analysis conducted in this study indicates a higher level of infiltration of monocytes, B cells, and plasma cells associated with Stat3, suggesting that in the immune microenvironment triggered by MIRI, Stat3 may participate in the formation of the vascular network by regulating immune cell infiltration.

Rela, also known as NF-κB3, MGC131774, and p65, is a prominent member among the five members of the NF-κB family. It possesses a distinctive transactivation domain and serves as an effective transcriptional activator for genes involved in the inflammatory response [35]. During MIRI, NF-κB is frequently activated, leading to the induction of inflammatory factors that exacerbate damage to myocardial cells. However, specific knockout of the RelA gene in animal models has demonstrated a decrease in both systemic ischemia-reperfusion injury and local injury resulting from branch coronary artery obstruction. Further investigations have revealed that the disruption of RelA restores the balance of calcium ions, which is disturbed by MIRI. This suggests that the protective effect of RelA on myocardial cells after ischemia-reperfusion may be linked to the regulation of intracellular calcium homeostasis [36]. Moreover, NF-κB activation following ischemia-reperfusion injury stimulates immune cells to produce pro-inflammatory molecules such as TNF-α and IL-1β, and it triggers injured tissues to secrete VEGF, thereby exerting a pro-angiogenic effect [37]. Immuno-infiltration analysis indicates that RelA exhibits higher levels of infiltration by monocytes and macrophages. In hypoxia-induced inflammatory mice, the supplementation of α-ketoglutarate significantly reduces the expression of RelA, subsequently decreasing monocyte and neutrophil counts and alleviating the inflammatory response [38]. IL-33, a major activator of macrophages, activates RelA through recognition by macrophages, resulting in the production of inflammatory factors associated with cellular damage and stress [39]. As the most abundant member of the NF-κB family, Rela may also contribute to the improvement of myocardial injury by participating in angiogenesis following MIRI.

Ubiquitination refers to the process of covalently attaching ubiquitin protein (Ub) to substrate proteins, thereby participating in post-translational modifications after protein translation. Ubiquitination serves a crucial regulatory role in various cellular processes, including signal transduction, transcription, apoptosis, autophagy, and other physiological and pathological activities. The gene Ubb, being a key determinant of cellular ubiquitination levels, plays a significant role in the regulation of protein degradation through the ubiquitin-proteasome pathway [40, 41]. Several studies have demonstrated that the ubiquitination-proteasome pathway directly influences downstream target gene hsRPB7 in the context of multi-organ tumor diseases. This pathway regulates angiogenesis and the development of vascular tumors by mediating the ubiquitination-induced degradation and reduction of vascular endothelial growth factor expression [42]. In the current study, immune-infiltration analysis revealed a noteworthy association between Ubb and the infiltration of plasma cells, T cells, and monocytes. Unfortunately, the research on Ubb in the context of MIRI remains limited. Nevertheless, investigations related to Immunoglobulin A Associated Vasculitis and renal ischemia-reperfusion injury have found that Ubb is involved in the regulation of immune cells, particularly in the immune microenvironment [43]. Considering that MIRI and angiogenesis are often accompanied by the release of inflammatory factors and alterations in the immune microenvironment, further in-depth research is essential to explore the potential significance of Ubb in angiogenesis and its role in MIRI.

Further analysis using CMap revealed that acitretin, derived from etretinate through a structural transformation from an ester to an acid structure, promotes normal keratinization by inhibiting abnormal proliferation and differentiation of keratinocytes. Additionally, avasimibe has the ability to regulate VEGF and participate in microvascular formation, thereby exerting its therapeutic effects [44]. Bortezomib, a novel proteasome inhibitor, has been shown to possess anti-inflammatory effects by inhibiting the activation of NF-κB, effectively alleviating angiotensin II-induced hypertension and aortic remodeling [45, 46]. In the context of myocardial I/R injury, bortezomib directly contributes to the inhibition of GRK2 degradation, thereby restoring the Akt pathway and protecting the injured myocardial tissue [47]. Genecard retrieval suggests that (4s)-5-Fluoro-L-Leucine has potential as a drug targeting Ubb. However, limited information is available regarding relevant experiments and clinical studies, indicating a relatively low clinical research value for (4s)-5-Fluoro-L-Leucine as a potential drug for Ubb.

In light of the aforementioned investigation, the substantial implications of three pivotal genes implicated in early vascular development—Stat3, Rela, and Ubb—are evident in the context of myocardial ischemia-reperfusion injury. Nevertheless, recognizing the constraints stemming from limited sequencing data and the inherent heterogeneity among distinct sequencing samples, our forthcoming research endeavors will focus on a comprehensive validation of the diagnostic relevance and clinical significance associated with these critical genes.

## 5. Conclusion

In conclusion, this study utilized bioinformatics analysis to identify three biomarkers, namely Stat3, Rela, and Ubb, which demonstrate a close association with angiogenesis and MIRI. The significance of these biomarkers was further confirmed through immune-infiltration analysis, ROC curve analysis, and single-cell sequencing platforms. Furthermore, we conducted molecular docking to predict potential small molecular compounds that target these hub genes and explored their binding sites. Nevertheless, additional validation and research are necessary, involving both in vitro and in vivo experiments, to determine the viability of Stat3, Rela, and Ubb as early therapeutic targets for angiogenesis in the pathological progression of MIRI.

## Supporting information

S1 File (ZIP)
